# RNA from stabilized whole blood enables more comprehensive immune gene expression profiling compared to RNA from peripheral blood mononuclear cells

**DOI:** 10.1371/journal.pone.0235413

**Published:** 2020-06-26

**Authors:** Fleur van der Sijde, Yunlei Li, Rick Schraauwen, Willem de Koning, Casper H. J. van Eijck, Dana A. M. Mustafa

**Affiliations:** 1 Department of Surgery, Erasmus MC, University Medical Center Rotterdam, Rotterdam, The Netherlands; 2 Department of Pathology, Clinical Bioinformatics Unit, Erasmus MC, University Medical Center Rotterdam, Rotterdam, The Netherlands; 3 Department of Pathology, Tumor Immuno-Pathology Laboratory, Erasmus MC, University Medical Center Rotterdam, Rotterdam, The Netherlands; European Institute of Oncology, ITALY

## Abstract

Monitoring changes in the immune profile in blood samples can help identifying changes in tumor biology and therapy responsiveness over time. Immune-related gene expression profiles offer a highly reproducible method to monitor changes of the immune system. However, measuring gene expression profiles in whole blood samples can be complicated because of the high protein and enzyme abundancy that affect the stability and quality of the RNA. Peripheral blood mononuclear cells (PBMCs) are one the most commonly used source for immune cell RNA extraction, though, this method does not reflect all components of the peripheral blood. The aim of this study was to determine the differences in immune-related gene expression between RNA isolated from stabilized whole blood and RNA isolated from PBMCs. Whole blood samples from 12 pancreatic cancer patients were collected before and after chemotherapy (*n* = 24). Blood samples were collected in both EDTA tubes, and Tempus tubes containing an RNA stabilizer (total *n* = 48). PBMCs were isolated from EDTA samples using Ficoll and were snap frozen. Subsequently, immune-related gene expression was profiled using the PanCancer Immune Profiling Panel of NanoString technology. Gene expression profiles of PBMCs were compared to that of Tempus tubes using the Advanced Analysis module of nSolver software. Both types of samples provided good quality RNA and gene expression measurements. However, RNA isolated from Tempus tubes resulted in significantly higher gene counts than PBMCs; 107/730 genes were exclusively detected in Tempus samples, while under the detection limit in PBMCs. In addition, 192/730 genes showed significantly higher gene counts in Tempus samples, 157/730 genes showed higher gene counts in PBMCs. Thus, RNA isolated from whole blood stabilizing blood tubes, such as Tempus tubes, enable higher gene counts and more comprehensive measurements of gene expression profiles compared to RNA isolated from PBMCs.

## Introduction

In this era of rapidly evolving immune monitoring and immune therapies in many different research fields, longitudinal peripheral blood immune profiling is becoming crucial to monitor the outcome of patients [1]. Peripheral blood sampling is a low-risk, non-invasive procedure and available at low costs [2]. Peripheral blood is the main route for transportation of immune cells, which thereby provides the opportunity to monitor the activity of the immune system [3, 4]. Monitoring the immune system requires more than profiling subtypes of immune cells. Blood-based gene expression profiles reflect activity of the immune response, including all types of circulating immune cells, their secreted cytokines, chemokines, and neoantigens.

Peripheral blood mononuclear cells (PBMCs) are one of the most commonly used sources of RNA to define the immune response in research [5]. Isolation of PBMCs provides a pellet of immune cells which can be stored after snap freezing to preserve the RNA. However, this pure mononuclear cell pellet mainly consists of lymphocytes [6], while other immune cells, such as erythrocytes, platelets and granulocytes are completely washed out [7]. All circulating RNA and secreted factors are lost during the various washing steps to purify PBMCs. Additionally, PBMC isolation is a time-consuming procedure and the method varies between laboratories [8]. On the other hand, whole blood represents the complete range of immune cells and their secreted factors and is easy to obtain. However, whole blood contains several proteins, enzymes, and RNases that prevent reliable isolation of RNA [9]. Collecting whole blood in tubes containing an RNA stabilizing reagent could provide a solution to this undesired phenomenon [10, 11]. Using an RNA stabilizer enables isolation of RNA from all blood components and secreted factors, instead of mononuclear cells only. In addition, such samples capture expression profiles that accurately reflect the transcriptome at time of blood collection with minimum sample handling artifacts. However, blood samples will be diluted with the stabilizing reagent and the percentage of RNA isolated from lymphocytes might be lower compared to PBMC pellets.

In this study, we compared the abundancy of measured immune-related genes, sensitivity of detection and the range of identified immune cells between RNA isolated from PBMCs and RNA isolated from whole blood samples collected in Tempus tubes containing a stabilizing reagent.

## Material and methods

### Patient selection

Samples from 12 patients, participating in a prospective cohort study including repeated peripheral blood sampling, were used in this experiment. All patients were diagnosed with pancreatic cancer and treated with FOLFIRINOX chemotherapy. Blood samples were collected the day before start of treatment and after one cycle of chemotherapy. Both blood samples for PBMC extraction in EDTA tubes (*n* = 24) and whole blood samples in Tempus tubes (*n* = 24) and were collected within the same blood draw at each time point resulting in a total *n* = 48.

This study was approved by the medical ethics committee of the Erasmus University Medical Center Rotterdam (MEC-2018-087) and samples were collected with patients’ written informed consent.

### Blood sample processing

For isolation of PBMCs, whole blood was collected in 10 mL EDTA tubes (Becton Dickinson, Franklin Lakes, NJ, USA). Within a maximum of four hours after collection, PBMCs were extracted with Ficoll-Paque technique, using LeucoSep tubes (Greiner Bio-One, Kremsmünster, Austria). After isolation and cell count with the Countess II Automated Cell Counter (Invitrogen, Carlsbad, CA, USA), PBMCs were snap frozen and stored at -80°C until further use. On average, 4 mL of whole blood was used to store one vial of PBMCs.

Whole blood was collected in Tempus tubes (Applied Biosystems, Foster City, CA, USA), in which 3 mL of whole blood is stabilized with 6 mL of RNA stabilizing reagent, resulting in a total volume of 9 mL. Tubes were stored at -80°C within four hours after collection until further use.

### RNA isolation

Total RNA from PBMCs was isolated from approximately 8.4x10^5^–1.3x10^7^ cells using the RNeasy Mini isolation kit (Qiagen, Hilden, Germany). Total RNA from Tempus tubes was isolated from 400 μL of stabilized whole blood using the NucleoSpin RNA Blood isolation kit (Macherey-Nagel, Düren, Germany). If concentrations were not sufficient, RNA was again isolated from 800 μL of stabilized whole blood instead. RNA concentrations and quality were measured using the Agilent 2100 BioAnalyzer (Santa Clara, CA, USA). To correct for degradation of the RNA, the percentage of fragments of 300–4000 nucleotides was used to calculate the corrected concentrations.

### Gene expression analysis

Gene expression analysis was performed using the PanCancer Immune Profiling Panel from NanoString technologies. The panel contains probes of 730 immune-related genes and 40 housekeeping genes, representing 24 different immune cell types and common checkpoint inhibitors, covering both adaptive and innate immune response. For each sample, 200 ng of total RNA, with a maximum of 7 μL (>28.6 ng/μL), was used. Hybridization was performed at 65°C for 17 hours using a SimpliAmp Thermal Cycler (Applied Biosystems, Foster City, CA, USA). The nCounter Flex system (NanoString, Seattle, WA, USA) was used for sample preparation. Gene counting was performed by scanning 490 Fields of View.

### Statistical analysis

Gene expression profiles of RNA isolated from PBMCs collected in EDTA tubes were compared with RNA isolated from Tempus tubes. Raw gene counts were normalized using the most stable housekeeping genes from the panel selected by the geNorm algorithm [12], incorporated in the Advanced Analysis module of nSolver software (version 2.0, NanoString, Seattle, WA, USA). Background threshold was determined as the average count of the negative controls + 2 standard deviations. Genes that showed a count above the threshold were considered as detected genes. Gene counts of most genes were not normally distributed across samples, even after logarithmic transformation. Therefore differential expression of genes between PBMC and Tempus RNA was tested with Mann-Whitney U tests and Benjamin-Hochberg procedures were used to correct for multiple testing.

Differentially expressed (DE) genes were further analyzed with Gene Set Analysis (GSA) as part of the Advanced Analysis. It enables to connect DE genes based on their function, resulting in scores for various pathways and cell types. Pathways and cell types are predefined using specific gene sets, as described previously [13]. The definition of cell types using these gene sets were found to be strongly correlated to cell types measured by flow cytometry in different tissue types, including cancers [14–16]. Differences in pathway scores and cell type scores were tested with Mann-Whitney U tests and Benjamin-Hochberg procedures were used to correct for multiple testing.

Adjusted *P*-values <0.05 were considered statistically significant. Statistical analyses were performed with R software (version 3.6.1, R Foundation for Statistical Computing, Vienna, Austria), using the packages stats (version 3.6.1) and matrixStats (version 0.56.0).

## Results

### RNA quality

All 24 PBMC samples isolated from EDTA tubes yielded sufficient RNA (>28.6 ng/μL). Ten of the Tempus samples showed insufficient RNA yields and required another isolation using 800 μL whole blood. After a second isolation, all Tempus samples yielded sufficient RNA concentrations. Also after correction of the RNA concentration for RNA with length of 300–4000 nucleotides, all samples showed good enough quantity and quality for immune gene expression profile measurements.

### Gene expression

In total, 31 housekeeping genes were selected for normalization. An overview of these selected housekeeping genes with their average expression and variance can be found in S1 Table.

**Fig 1:**
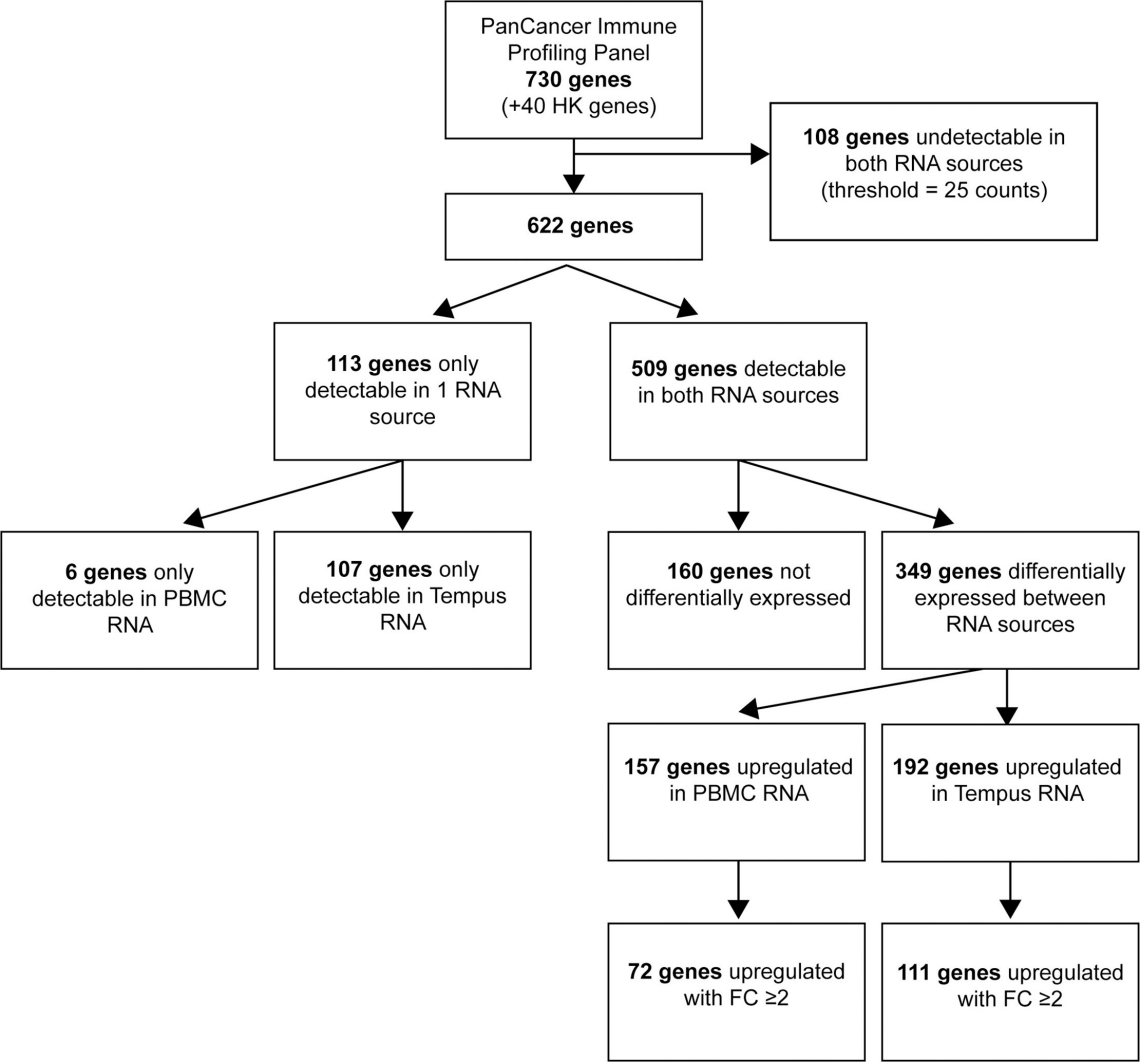
Diagram of genes detected or upregulated in one source of input RNA. Out of 700 detected genes, 107 were only detected in Tempus samples, six genes were only detected in PBMC samples. Most genes were upregulated in Tempus samples (192 genes), while 157 genes were upregulated in PBMC samples. HK = housekeeping.

After normalization, the average gene count in PBMC samples was 790.02 and in Tempus samples 1188.64 (*P*<0.001). Out of 730 genes, 108 were not detected in any group (PBMCs or Tempus); the median count of these genes was below the threshold of 25 counts, as determined by the negative controls. A total of 107 genes was exclusively detected in Tempus samples. In contrast, only six genes were exclusively detected in PBMCs samples. Moreover, 157 genes were significantly higher expressed in PBMC samples with *P*<0.05 (of which 72 genes with a fold of change ≥2), while 192 genes were significantly higher expressed in Tempus samples with *P*<0.05 (of which 111 genes with a fold of change ≥2). A comprehensive flowchart can be found in Fig 1. The variation between PBMCs and Tempus samples per patient is visualized in S1 Fig. 21/24 patients show the same trend as in the total group: the number of detectable genes and the number of exclusively detectable genes were higher in Tempus samples compared to PBMC samples.

**Fig 2:**
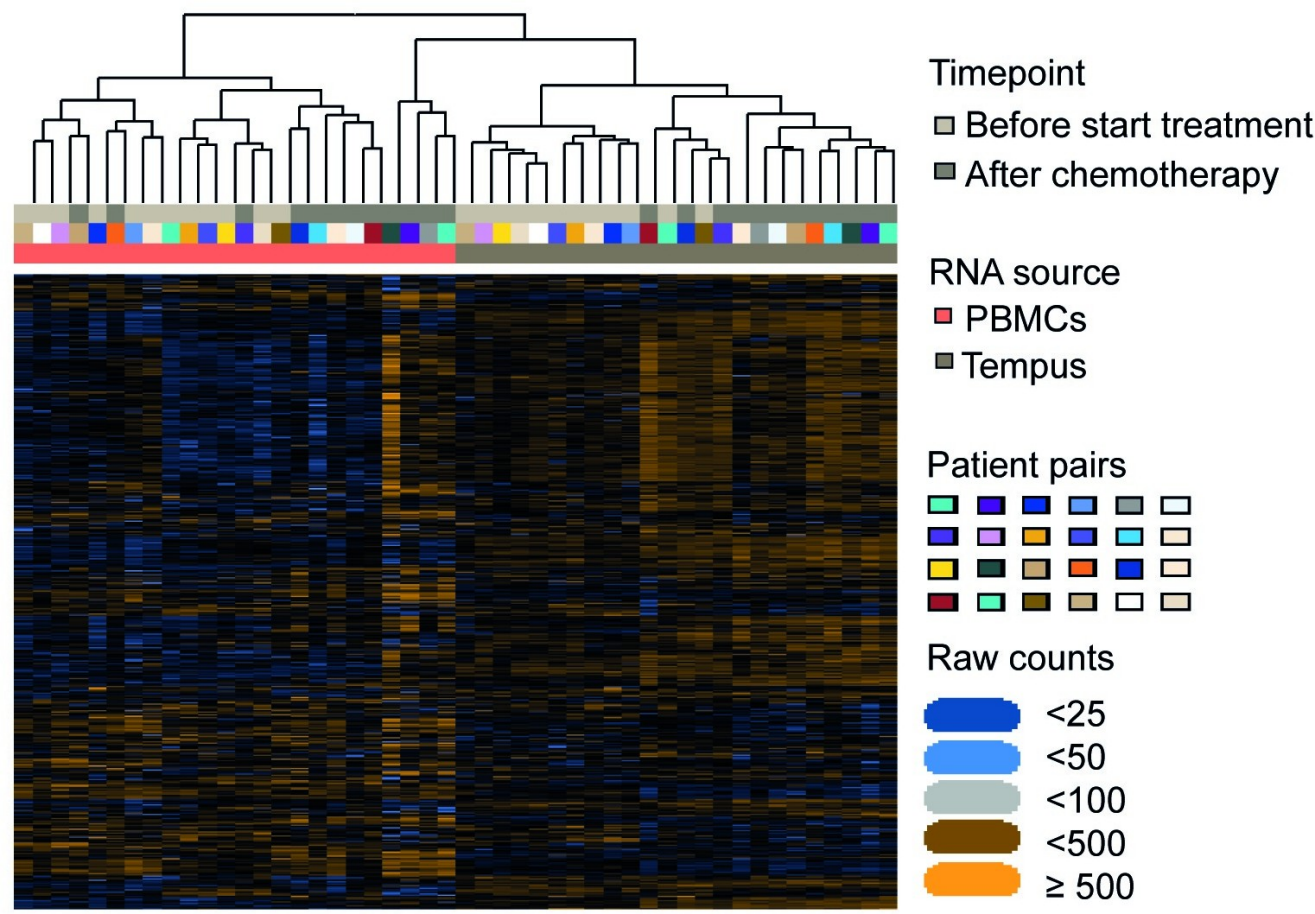
Heatmap of normalized data. Unsupervised clustering of PBMCs and Tempus samples resulted in a perfect clustering based on the sample type.

Unsupervised clustering for all genes resulted in a perfect clustering of PBMC samples and Tempus samples (Fig 2), highlighting that the preservation method affects the expression of immune-related genes more than the biological variation between samples.

### Pathway scores

**Fig 3:**
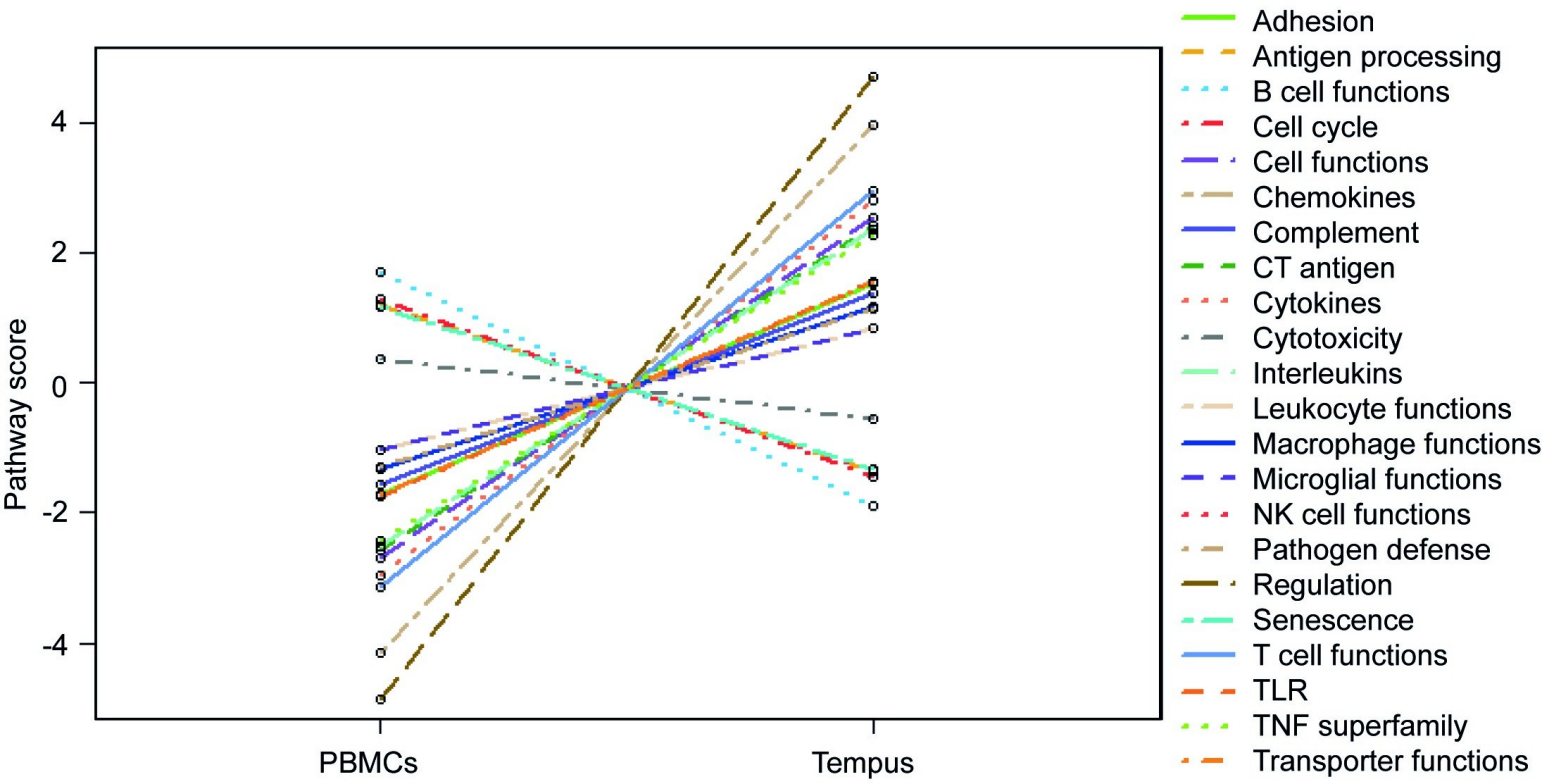
Relative expression of pathway scores between PBMCs and Tempus RNA. Six pathways (Antigen Processing, B cell functions, Cell Cycle, Cytotoxicity, NK cell functions, and Senesence) are upregulated in PBMC samples compared to Tempus samples. All other pathways (22) are upregulated in Tempus, reflecting the higher expression of detected genes in Tempus samples compared to PBMCs.

All pathway scores were significantly different between PBMCs and Tempus samples (Fig 3, Table 1). The pathways Antigen Processing (*P*<0.001), B cell Functions (*P*<0.001), Cell Cycle (*P*<0.001), Cytotoxicity (*P* = 0.014), NK cell Functions (*P*<0.001), and Senescence (*P*<0.001) scored higher in PBMCs, while all other pathways scored higher in Tempus samples with *P*<0.001 (Table 1). Similar to the results of gene expression, PBMCs and Tempus samples clustered perfectly based on pathway scoring (S2 Fig). However, some pathway scores are based on genes that were hardly detected in PBMC samples. For example, the Cancer/Testis Antigen (CT Antigen) pathway is defined by 12 genes of which only one gene was detected in PBMC samples but 11 were detected in Tempus samples. In addition, Complement pathway is defined by 15 genes, all of them were detected in Tempus samples, but only 12 were detected in PBMC samples. For the Interleukin pathway, the majority of genes, 25/38, was not detected in PBMCs. Also in the Chemokine pathway almost half of the defining genes, 42 out of 99, could not be detected in PBMC samples. In all pathways, less defining genes were detected in PBMC samples compared to Tempus samples.

**Table 1:** Comparison of relative pathway scores between peripheral blood mononuclear cells (PBMCs) and Tempus samples.

Pathway	Number of defining genes (not detected)	Score PBMCs, median (IQR)	Score Tempus, median (IQR)	Adjusted *P*-value
Adhesion	25 (PBMC: 6, Tempus: 4)	-1.85 (-2.97 - -0.70)	2.02 (0.09–3.16)	<0.001
Antigen Processing	22 (PBMC: 2, Tempus: 2)	1.10 (0.35–2.19)	-1.42 (-2.26 - -0.64)	<0.001
B cell Functions	25 (PBMC: 6, Tempus: 4)	1.99 (1.18–2.34)	-1.87 (-2.64 - -1.22)	<0.001
Cell Cycle	13 (PBMC: 1)	1.70 (0.77–2.05)	-1.39 (-1.59 - -1.18)	<0.001
Cell Functions	71 (PBMC: 22, Tempus: 14)	-3.16 (-3.84 - -2.20)	3.17 (1.16–4.17)	<0.001
Chemokines	99 (PBMC: 42, Tempus: 21)	-5.15 (-5.78 - -3.55)	4.27 (2.14–6.22)	<0.001
Complement	15 (PBMC: 3)	-1.88 (-2.71 - -1.04)	1.17 (0.65–2.59)	<0.001
CT Antigen	12 (PBMC: 11, Tempus: 1)	-3.50 (-4.62 - -0.89)	2.32 (1.35–4.31)	<0.001
Cytokines	56 (PBMC: 19, Tempus: 12)	-3.78 (-4.28 - -2.39)	2.96 (1.38–4.41)	<0.001
Cytotoxicity	10	0.76 (0.09–1.12)	-0.34 (-1.44–0.46)	0.014
Interleukins	38 (PBMC: 25, Tempus: 13)	-3.25 (-4.16 - -1.50)	2.12 (1.24–4.20)	<0.001
Leukocyte Functions	8	-1.13 (-1.98 - -0.21)	1.12 (0.06–1.78)	<0.001
Macrophage Functions	15 (PBMC: 1)	-1.41 (-1.80 - -0.83)	1.34 (0.68–1.98)	<0.001
Microglial Functions	5 (PBMC: 1)	-0.90 (-1.18 - -0.58)	0.95 (0.66–1.40)	<0.001
NK Cell Functions	31 (PBMC: 6, Tempus: 2_	1.56 (0.74–2.30)	-1.70 (-2.62–0.12)	<0.001
Pathogen Defense	12 (PBMC: 2)	-1.59 (-1.94 - -0.77)	1.25 (0.20–2.50)	<0.001
Regulation	155 (PBMC: 36, Tempus: 17)	-5.32 (-5.96 - -4.16)	5.27 (3.00–6.82)	<0.001
Senescence	12 (PBMC: 1, Tempus: 1)	1.25 (1.03–1.50)	-1.28 (-1.46–1.15)	<0.001
T cell Functions	70 (PBMC: 18, Tempus: 11)	-3.23 (-4.05 - -2.31)	3.42 (1.52–4.70)	<0.001
TLR	11 (PBMC: 2)	-1.53 (-2.18 - -1.07)	1.98 (0.69–2.67)	<0.001
TNF Superfamily	30 (PBMC: 9, Tempus: 8)	-2.33 (-2.87 - -1.96)	2.50 (1.99–2.90)	<0.001
Transporter Functions	22 (PBMC: 3, Tempus: 2)	-1.83 (-2.21 - -1.31)	1.73 (1.18–2.28)	<0.001

*P*-values are calculated with Mann-Whitney U tests and adjusted with Benjamin-Hochberg procedure. IQR = interquartile range, TLR = toll-like receptor, CT antigen = cancer/testis antigen, NK = natural killer, TNF = tumor necrosis factor.

As presented in S1 Fig, in all samples more pathways are overexpressed in Tempus than there are pathways overexpressed in PBMC samples.

### Cell type scores

**Fig 4:**
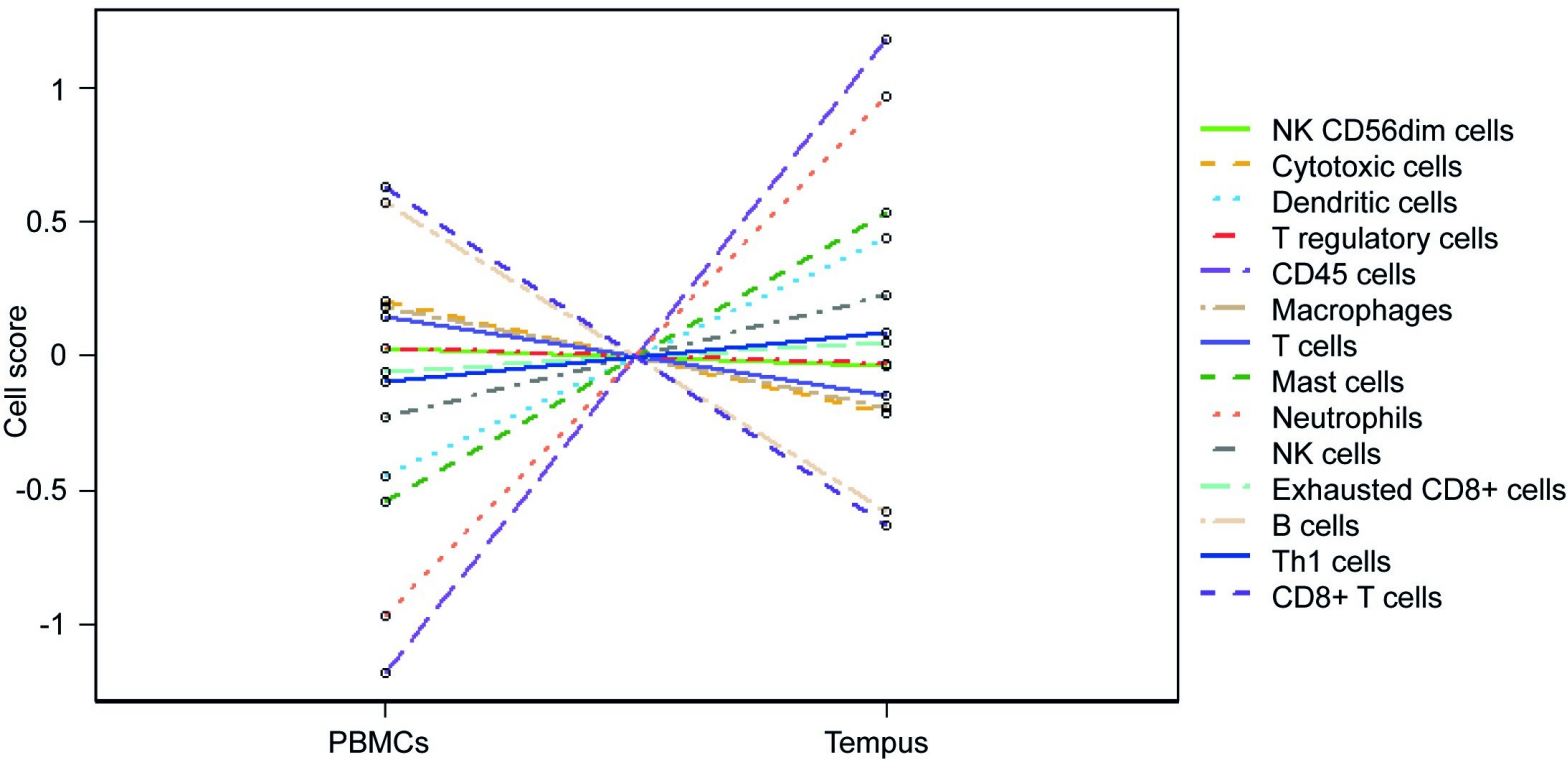
Relative expression of cell types between PBMCs and Tempus RNA. Dendritic cells, CD45+ cells, mast cells, neutrophils, and natural killer (NK) cells show higher scores in Tempus samples. Cytotoxic cells, macrophages, B cells and CD8+ T cells were detected at a higher level in PBMCs.

Expression of gene sets that are only expressed by one immune cell type were used to create a cell type score (S2 Table). The average cell type score was compared between PBMCs and Tempus samples (Table 2). The following cell types were found to be relatively higher in Tempus preservation methods: dendritic cells (*P*<0.001), CD45+ cells (*P*<0.001), mast cells (*P*<0.001), neutrophils (*P*<0.001), natural killer cells (*P* = 0.022). However, cytotoxic cells (*P* = 0.024), macrophages (*P* = 0.009), B cells (*P*<0.001), and CD8+ T cells (*P*<0.001) were relatively higher in PBMC samples. Relative expression of cell types is visualized in Fig 4.

**Table 2:** Comparison of cell scores between peripheral blood mononuclear cells (PBMCs) and Tempus samples.

Cell type	Number of defining genes (not detected)	Score PBMCs, median (IQR)	Score Tempus, median (IQR)	Adjusted *P*-value
NK CD56dim cells	2	5.71 (5.25–6.27)	5.61 (5.26–6.10)	0.633
Cytotoxic cells	9	9.54 (9.27–9.86)	9.14 (8.39–9.44)	0.024
Dendritic cells	3 (PBMC: 3, Tempus: 2)	3.54 (3.34–3.89)	4.65 (4.14–5.06)	<0.001
Regulatory T cells	1	5.08 (4.65–5.75)	5.06 (4.74–5.50)	0.675
CD45+ cells	1	10.69 (10.48–11.14)	13.26 (12.93–13.71)	<0.001
Macrophages	2	9.10 (8.58–9.43)	8.53 (8.34–8.70)	0.009
T cells	5	8.11 (7.97–8.76)	7.95 (7.55–8.41)	0.136
Mast cells	2 (PBMC: 1)	4.47 (4.16–5.18)	5.81 (5.51–6.05)	<0.001
Neutrophils	2	11.85 (11.12–12.46)	14.38 (12.82–15.09)	<0.001
Natural killer cells	2	6.52 (6.30–7.34)	7.28 (6.79–7.44)	0.022
Exhausted CD8+ cells	2	6.40 (6.02–6.61)	6.36 (6.13–6.71)	0.633
B cells	3	7.72 (7.11–8.33)	6.64 (6.37–6.92)	<0.001
Th1 cells	1	8.05 (7.64–8.48)	8.19 (7.81–5.78)	0.548
CD8+ T cells	2	7.96 (7.34–8.40)	6.55 (6.12–7.07)	<0.001

Data are presented as log2 values. *P*-values are calculated with Mann-Whitney U tests and adjusted with Benjamin-Hochberg procedure. IQR = interquartile range, Th = T-helper.

The definition of dendritic cells is based on the expression of three genes that all showed a median count below the negative threshold in PBMC samples. In Tempus samples, two of the genes showed an insufficient median gene count below the threshold. For mast cells, one out of two defining genes was not detected in PBMC samples, while detectable in Tempus samples.

In 8/24 samples more cell types were overexpressed in Tempus. In 16/24 samples more cell types were overexpressed in PBMCs (S1 Fig).

## Discussion

The aim of this study was to determine the most reliable method of RNA blood cell extraction for monitoring of the immune system by immune-related gene expression analysis. Although both RNA preservation methods resulted in good quality and quantity of RNA, gene expression profiles revealed a significant variation between PBMCs and Tempus samples. Nevertheless, gene expression profiles of both sample types showed promising results with a sufficient number of detected genes. To our knowledge, this is the first study to compare gene expression profiles of RNA isolated from PBMCs and RNA isolated from whole blood stabilizing tubes using NanoString technology.

Using an RNA stabilizing reagent preserves gene activity and enables detection of a higher number of immune-related genes from whole blood samples compared to PBMC RNA. A total of 127 genes was detected exclusively in Tempus samples, while only six genes were detected exclusively in PBMC samples. Overall, average gene counts were 51% higher in Tempus samples compared to PBMCs. Higher gene counts enable a more reliable and comprehensive analysis, because the genes are certainly above the threshold. Using an RNA stabilizing reagent ensures that RNA from all sources is preserved, including RNA from all blood components and secreted RNA that is washed away when purifying PBMCs from EDTA tubes. For example, granulocytes are preserved in Tempus tubes, therefore the genes that define them showed higher counts in Tempus compared to PBMCs (Table 2). In addition, the time consumed between drawing blood samples from patients, isolation of PBMCs, and storage at -80°C is significantly longer (1–3 hours) than drawing blood in Tempus tubes that do not need any processing before storage. During blood processing and PBMC isolation, RNA is exposed to various enzymes resulting in RNA degradation, affecting the most vulnerable and low abundant dendritic cells first [17]. As a result, not only genes from non-mononuclear cells, but also genes associated with mononuclear cells, for example dendritic cells and natural killer cells, were measured at higher counts in Tempus samples compared to PBMCs. We hypothesized that enrichment of mononuclear cells in PBMCs would lead to detection of genes expressed by mononuclear cells at a higher level. However, in this study only a minority of genes was expressed at a higher level in PBMCs. On the other hand, blood collected in Tempus tubes is diluted with a stabilizing reagent, which might cause lower gene expression results, hence the relative higher counts of 157 genes in PBMCs compared to Tempus samples.

Unsupervised clustering showed perfect clustering of PBMC and Tempus samples, highlighting that the RNA preservation method should be chosen carefully when profiling RNA of blood samples. Therefore, measuring the gene expression profiles of RNA samples isolated from various blood tubes or sources in one experiment is not recommended. Although PBMCs and whole blood stored in Tempus tubes showed similar gene expression profiles, absolute gene expression levels did significantly differ, which was also visualized by clustering of samples by the type of RNA source. Similar results were found by Rollins et al. using Affymetrix arrays [18].

As a consequence of the detection and gene count variation between PBMCs and Tempus samples, unsupervised clustering based on the pathway scores showed a perfect separation between the samples. Most pathways were upregulated in Tempus tubes compared to PBMCs, which is explained by the higher cell counts of all defined pathways. The average count of genes would not affect the pathway scores when samples of the same RNA source will be used in one experiment. The higher gene counts and therefore the scores of pathways are influenced by preservation of RNA with the stabilizing reagent. In addition, some pathway scores are based on genes that were not detected in PBMC samples. For example, the CT Antigen pathway is defined by 12 genes, of which only one gene was detected in the majority of PBMC samples. However, all 12 genes were detected in Tempus samples, resulting in a higher score of the CT Antigen pathway in Tempus tubes. Another example is the Complement pathway, which is defined by 15 genes. All 15 were detected in Tempus samples, while only 12 genes were detected in PBMCs samples, which reflects the higher score for Complement pathway in Tempus samples. At least the CT Antigen pathway should be excluded from analysis in experiments when PBMC samples are used, because it is impossible to determine if the genes are not present or if they are under the detection limit. As another example, nine genes (out of 11) defined as the TLR pathway were detected in all PBMC and Tempus samples, which should be sufficient to accurately score the pathway.

Granulocytes (neutrophils, mast cells) were not the only cells upregulated in Tempus tubes, but also mononuclear cells (e.g. dendritic cells), for which was enriched in PBMC samples. However, dendritic cell genes were all under the detection limit in PBMC samples. For mast cells only one out of two genes could be detected in PBMCs. In order to detect genes defining dendritic cells or mast cells, an RNA stabilizing reagent is needed to preserve the activity of these genes. Dendritic cells are known to represent only a very small percentage of the total blood cell count in the circulation [6, 17]. Their genes are vulnerable and could probably therefore not be detected in PBMCs. The definition of cells in nSolver software is limited by the number of genes used to define the cells. T helper 1 (Th1), regulatory T cells, and CD45+ cells are all based on the expression of one gene, therefore, the scores of these cell types needs to be confirmed. The other types of cells that were defined using multiple genes are more reliable (S2 Table). Quality controlling the data used in cell type definition and pathway scoring is extremely important in any experiment using NanoString technology. The fact that CD45+ cells are detected at a higher cell count in Tempus compared to PBMCs, even though in Tempus relatively less CD45+ cells are present and thus less RNA expression of the CD45 gene *PTPRC* is present, confirms our conclusion that RNA is preserved better in Tempus.

Besides the better RNA preservation, there are additional advantages of using stabilized whole blood samples, among which is the possibility to detect non-mononuclear cells. The immune system is not dependent on mononuclear cells alone, and other cells, such as granulocytes, are extremely important to investigate. The quantity and function of immune cells, including mononuclear cells, often depends on the presence and interaction with other types of immune cells.

However, there are advantages and disadvantages for both sources of RNA for measuring immune-related gene expression. Collecting whole blood in RNA stabilizing tubes is convenient and does not require extra processing before storage. PBMC extraction from EDTA tubes, however, is a time-consuming method (±1 hour when using LeucoSep tubes for easy separation of PBMCs), and isolation protocols vary between researchers and laboratories. To reach sufficient expression data, PBMCs isolation from at least 4 mL of whole blood is necessary for RNA extraction. In Tempus tubes, extra RNA extractions using a higher amount, of input material (800 μL instead of 400 μL) was in this experiment necessary in 10/24 samples to reach sufficient RNA yields to perform multiplex measurements. However, this resulted in a maximum of 400 μL undiluted whole blood used. An overview of pros and cons are summarized in Table 3. Whether the type of RNA preservation tube (e.g. Tempus versus PAXgene) affects RNA quality and expression is not clear yet. There is evidence that differences between these tubes have influence on absolute gene expression levels, but this effect might be overcome by modification of the isolation protocol [4, 19].

**Table 3:** Pros and cons of using RNA from PBMCs or Tempus using NanoString immunology gene expression analysis.

*Pros PBMC RNA*
Enriched RNA from mononuclear cells
*Cons PBMC RNA*
At least 4 mL of whole blood necessary
Relatively long hands-on time for cell extraction until freezing of cells
Important immunology related genes might not be detected due to the source of RNA (not mononuclear cells)
Variability of isolation methods in different laboratories
*Pros Tempus RNA*
Only 3 mL of whole blood necessary, which can be used for multiple RNA extractions. On average, only 800 μL of stabilized whole blood (= 267 μL of whole blood + 533 μL of stabilizing reagent) is needed per extraction.
No cell extraction. Therefore, no hands- on time needed to process the samples.
RNA preservation with stabilizing reagent
RNA from all blood components and free RNA are included.
Higher number of genes detected
Higher average gene count
*Cons Tempus RNA*
Relatively expensive collection tubes (€7.06/tube)
Dilution of immune cells and their RNA by stabilizing reagent
Multiple RNA isolations might be necessary to reach the desired concentration of RNA

Although not included in this experiment, differences in measured gene expression between RNA sources are probably not limited to NanoString technologies, but should also be considered in other types of gene expression analysis.

In conclusion, RNA isolated from PBMCs extracted from EDTA tubes and RNA isolated from Tempus tubes resulted in good RNA quality and quantity that enabled gene expression profiling with NanoString technology. Gene expression data were mostly affected by the RNA preservation method. Although profiling immune-related genes using RNA isolated from PBMCs provided sufficient gene counts and measurements, using RNA from stabilized whole blood, in this case collected in Tempus tubes, resulted in higher gene counts and thus more comprehensive measurements. Therefore, we do not recommend combining various sample types within the same experiment. In particular, certain cell types and pathways (dendritic cells, and Complement pathway) cannot be reliably measured when using PBMC samples.

## Supporting information

S1 Fig Amount of detected genes, and overexpressed pathways and cell types for all samples individually. In most samples, Tempus provides more detected genes and more genes that are exclusively detected compared to PBMCs. In all samples, more pathways are overexpressed in Tempus compared to PBMCs. Only in 8/24 samples more cell scores are overexpressed in Tempus compared to PBMCs.
(TIF)

S2 Fig Heatmap of pathway scores. There is perfect clustering of PBMC and Tempus samples based on relative pathway scoring.
(TIF)

S1 Table Housekeeping genes selected for normalization, with their average expression and variance. (DOCX)

S2 Table Genes used to define cell types. (DOCX)
