# Supplementary figures and images for: RNA from stabilized whole blood enables more comprehensive immune gene expression profiling compared to RNA from peripheral blood mononuclear cells
Source: PLoS One. 2020 Jun 26;15(6):e0235413. doi: 10.1371/journal.pone.0235413 (PMC7319339; doi:10.1371/journal.pone.0235413)

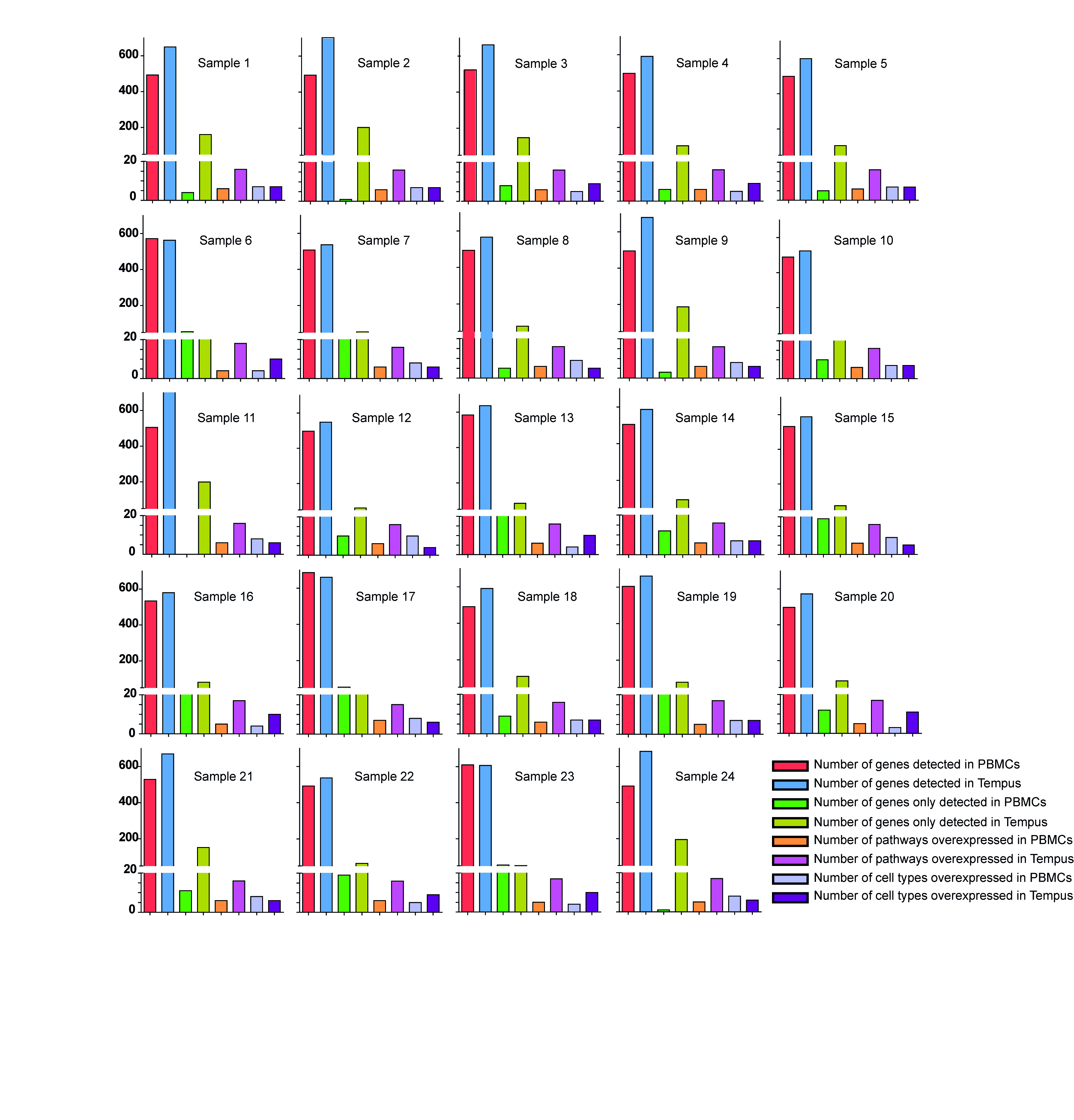

Supplement: S1 Fig — In most samples, Tempus provides more detected genes and more genes that are exclusively detected compared to PBMCs. In all samples, more pathways are overexpressed in Tempus compared to PBMCs. Only in 8/24 samples more cell scores are overexpressed in Tempus compared to PBMCs. (TIF) [file pone.0235413.s001.tif]

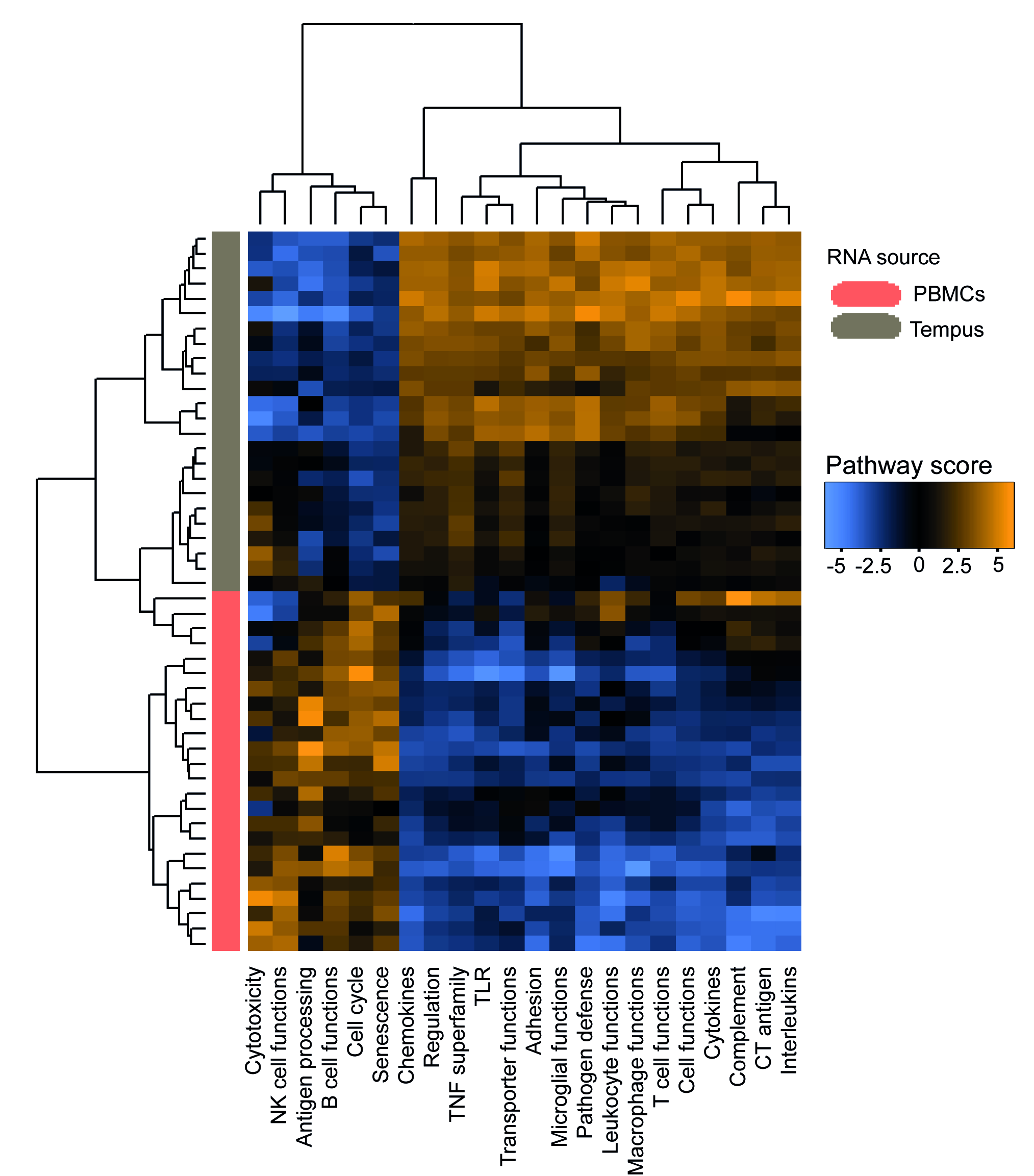

Supplement: S2 Fig — There is perfect clustering of PBMC and Tempus samples based on relative pathway scoring. (TIF) [file pone.0235413.s002.tif]
