# Supplementary material for: RNA from stabilized whole blood enables more comprehensive immune gene expression profiling compared to RNA from peripheral blood mononuclear cells
Source: PLoS One. 2020 Jun 26;15(6):e0235413. doi: 10.1371/journal.pone.0235413 (PMC7319339; doi:10.1371/journal.pone.0235413)
Supplement: S1 Table — (DOCX) [file pone.0235413.s003.docx]

| **Gene** | **Mean expression (log2)** | **Variance (log2)** |
| --- | --- | --- |
| *TBP* | 7.91 | 0.03 |
| *EIF2B4* | 7.70 | 0.03 |
| *PRPF38A* | 9.01 | 0.03 |
| *HDAC3* | 7.78 | 0.04 |
| *ABCF1* | 7.93 | 0.09 |
| *MRPS5* | 8.82 | 0.09 |
| *SF3A3* | 8.43 | 0.15 |
| *DNAJC14* | 6.59 | 0.12 |
| *EDC3* | 7.57 | 0.18 |
| *SDHA* | 7.76 | 0.15 |
| *COG7* | 7.24 | 0.16 |
| *ERCC3* | 6.84 | 0.17 |
| *TLK2* | 8.28 | 0.16 |
| *CNOT4* | 7.81 | 0.14 |
| *NOL7* | 8.20 | 0.09 |
| *DHX16* | 7.16 | 0.13 |
| *AGK* | 6.74 | 0.11 |
| *DDX50* | 7.39 | 0.13 |
| *HPRT1* | 8.26 | 0.11 |
| *ZC3H14* | 6.95 | 0.12 |
| *PPIA* | 7.34 | 0.23 |
| *SAP130* | 7.77 | 0.09 |
| *NUBP1* | 5.87 | 0.16 |
| *AMMECR1L* | 7.10 | 0.15 |
| *ZNF346* | 5.79 | 0.16 |
| *TUBB* | 8.86 | 0.23 |
| *FCF1* | 9.41 | 0.23 |
| *ZKSCAN5* | 4.94 | 0.22 |
| *TMUB2* | 8.13 | 0.24 |
| *ZNF143* | 8.26 | 0.28 |
| *TRIM39* | 6.40 | 0.30 |
