# Supplementary material for: RNA from stabilized whole blood enables more comprehensive immune gene expression profiling compared to RNA from peripheral blood mononuclear cells
Source: PLoS One. 2020 Jun 26;15(6):e0235413. doi: 10.1371/journal.pone.0235413 (PMC7319339; doi:10.1371/journal.pone.0235413)
Supplement: S2 Table — (DOCX) [file pone.0235413.s004.docx]

| **Cell type** | **Definition genes** | ***P*-value** |
| --- | --- | --- |
| NK CD56dim cells | *IL21R*  *KIR3DL2*  *KIR3DL1* (discarded)  *KIR_Inhibiting_Subgroup_2* (discarded) | 0.63 |
| Cytotoxic cells | *GZMB*  *PRF1*  *KLRK1*  *GZMH*  *KLRB1*  *KLRD1*  *GNLY*  *GZMA*  *CTSW* | 0 |
| Dendritic cells | *CD209*  *HSD11B1*  *CCL13* | 0.04 |
| Regulatory T cells | *FOXP3* | NA |
| CD45+ cells | *PTPRC* | NA |
| Macrophages | *CD163*  *CD68*  *CD84* (discarded) | 0.40 |
| T cells | *CD3G*  *SH2D1A*  *CD6*  *CD3D*  *CD3E* | 0 |
| Mast cells | *TPSAB1*  *MS4A2* | 0.29 |
| Neutrophils | *CSF3R*  *S100A12* | 0.02 |
| Natural killer cells | *XCL2*  *NCR1* | 0.50 |
| Exhausted CD8+ cells | *CD244*  *EOMES*  *LAG3* (discarded) | 0.60 |
| B cells | *BLK*  *CD19*  *MS4A1*  *TNFRSF17* (discarded) | 0.02 |
| Th1 cells | *TBX21* | NA |
| CD8+ T cells | *CD8A*  *CD8B* | 0.01 |

The *P*-value is calculated as the proportion (out of 1000 random gene sets) of gene sets that shows a better correlation than the gene sets defined by NanoString.
